# The application value and challenges of metagenomic next-generation sequencing in the diagnosis of periprosthetic joint infection after arthroplasty

**DOI:** 10.3389/fmed.2025.1686503

**Published:** 2025-10-02

**Authors:** Huahui Huang, Yan Tong, Xiunian Hu, Fa-ke Liao, Rijiang Chen

**Affiliations:** Longyan First Affiliated Hospital of Fujian Medical University, Longyan, China

**Keywords:** mNGS, PJI, infection, culture-negative, diagnostic accuracy

## Abstract

Metagenomic next-generation sequencing (mNGS) demonstrates high sensitivity, rapid diagnostic capabilities, and the potential to identify complex pathogens in periprosthetic joint infection (PJI) following arthroplasty, particularly when conventional culture methods are limited. mNGS enables the detection of polymicrobial infections and rare/fastidious pathogens, along with the ability to predict antimicrobial resistance (AMR) genes; however, the concordance between genotypic predictions and phenotypic resistance profiles requires further validation. In clinical practice, mNGS overcomes biofilm-related diagnostic barriers, facilitating early targeted antibiotic therapy and potentially reducing unnecessary revision surgeries, thereby lowering overall healthcare costs and improving patient outcomes. Nevertheless, its widespread adoption is hindered by high costs, lack of standardization, and risks of false-positive/false-negative results. Future research priorities include optimizing sample processing protocols, host DNA depletion, establishing diagnostic thresholds, and validating mNGS through integration with conventional methods. This review synthesizes recent advances in the diagnostic accuracy and clinical utility of mNGS for PJI, aiming to provide evidence-based insights for therapeutic decision-making and enhance the prevention and management of PJI.

## 1 Introduction

Periprosthetic joint infection (PJI) following arthroplasty remains one of the most formidable complications in joint replacement surgery (1), with its incidence and mortality rates demonstrating a progressive annual increase (2–4). PJI not only imposes severe physiological and psychological burdens on patients but also substantially elevates healthcare costs and socioeconomic expenditures (5–8). The diversity and inconsistent application of diagnostic criteria for PJI represent a major challenge in current clinical practice and research. The JS-BACH classification system categorizes patients into “uncomplicated,” “complex,” and “limited treatment options” based on five dimensions: joint specificity, extent of bone involvement, antibiotic options, soft tissue coverage, and host status. This framework comprehensively covers key factors influencing PJI prognosis (9, 10). Although this classification shows promise in initial studies, its external validity and generalizability require further validation (9). In contrast, the EBJIS criteria classify PJI into “confirmed,” “likely,” and “unlikely,” relying primarily on microbiological evidence, histopathology, synovial fluid analysis, and imaging results (11). These criteria perform particularly well in distinguishing chronic PJI, though their sensitivity depends on comprehensive microbiological testing (12, 13). Additionally, the diagnostic criteria proposed by MSIS are widely used for the clinical identification of PJI, yet their accuracy and applicability remain controversial (14). For example, in periprosthetic shoulder infection, the MSIS criteria may fail to reliably detect infection, with 30% of cases requiring diagnosis based on other clinical parameters (15). The use of different diagnostic standards leads to significant variation in reported PJI incidence rates (21–32%) (16, 17). In recent years, important progress has been made in the prevention, diagnosis, and treatment of PJI. Nevertheless, numerous challenges remain, such as antibiotic resistance due to biofilm formation, diversity of pathogens, lack of uniform diagnostic criteria, and the absence of personalized treatment strategies (18–20).

The reported incidence of PJI following primary arthroplasty is approximately 0. 4%, which rises to 1. 6% after revision surgery (21). Conventional pathogen detection methods suffer from low sensitivity, prolonged processing times, and limited microbial detection capabilities (22). This is particularly evident in culture-negative PJI (CN PJI), where approximately 48% of infections fail to yield identifiable pathogens via culture (23). Moreover, recent antibiotic use heightens the risk of false-negative results, exacerbating diagnostic challenges (24). The advent of metagenomic next-generation sequencing (mNGS) has recently emerged as a transformative strategy for precise PJI diagnosis.

## 2 Principles and optimization of mNGS technology

mNGS demonstrates significant advantages in infection diagnosis (25). By rapidly identifying pathogens and their genomic characteristics, mNGS provides timely and precise decision-making support for clinical treatment, thereby improving patient outcomes. Particularly in complex infections and public health emergencies, mNGS exhibits diagnostic value unmatched by conventional methods. The core principle of mNGS involves random fragmentation and sequencing of nucleic acids extracted from samples without prior knowledge of pathogens (25). Its workflow primarily includes: (1) sample processing; (2) nucleic acid extraction; (3) library preparation; (4) high-throughput sequencing; and (5) bioinformatic analysis. The greatest strength of this method lies in its ‘hypothesis-free’ detection capability, enabling unbiased identification of all microbial components in a sample, including bacteria, fungi, viruses, and rare pathogens (26). However, clinical application of mNGS still faces challenges, such as high costs, lack of standardized protocols, false-positive risks due to host nucleic acid interference (23), and the need for further optimization of detection workflows across different specimen types (27). Despite these limitations with continuous advancements in bioinformatics tools and sequencing technologies, mNGS is poised to become a vital tool in routine clinical diagnostics.

## 3 Diagnostic accuracy of mNGS for PJI

In recent years, mNGS has demonstrated transformative potential in the pathogen diagnosis of PJI, particularly serving as an effective tool for analyzing microbial communities in culture-negative and complex infections (28, 29). mNGS is being increasingly applied in detecting pathogens for periprosthetic joint infections (PJI), sepsis, and other infectious diseases (23). By synthesizing existing research, this article explores the unique value of mNGS in PJI to provide evidence-based guidance for clinical practice.

### 3.1 Comparative analysis of mNGS versus conventional culture methods

Culture-negative periprosthetic joint infection (CN-PJI) represents a major challenge in the diagnosis and treatment of PJI. As the gold standard, conventional microbial culture suffers from limitations including low sensitivity, time-consuming procedures, susceptibility to antibiotic interference, and inability to identify causative pathogens (24, 30), posing significant challenges for clinicians when formulating treatment strategies.

Multiple studies demonstrate that mNGS exhibits significantly higher overall sensitivity than conventional culture (31), particularly in CN PJI, where it detects additional rare pathogens missed by traditional methods (32, 33). However, mNGS may still yield false-negative results, potentially overlooking certain culture-positive pathogens (34). Implementing a “clinician-microbiologist-bioinformatician” tripartite consultation model could mitigate diagnostic inaccuracies. Notably, mNGS detects polymicrobial infections at 1. 5 × the rate of culture, yet its relatively low specificity (60%) necessitates caution against overdiagnosis (35). Among specimen types, sonicate fluid from prosthetic devices shows superior pathogen detection rates via mNGS (27, 35), attributable to its capacity to liberate biofilm-embedded microbes, yielding >10-fold higher sequencing reads and >5-fold greater genome coverage compared to other samples (36, 37). Current technical limitations center on host DNA contamination and bioinformatic complexity, mandating standardized thresholds (e. g., pathogen-specific read counts) to enhance reliability. Furthermore, traditional molecular methods like 16S rDNA PCR concord with mNGS in only 73%-86. 5% of cases, underscoring the necessity of integrating clinical judgment to avoid overreliance on any single technique (38, 39) (Table 1).

**Table 1:** Sensitivity and specificity of mNGS.

Sensitivity and specificity	mNGS	Cultures
Ivy et al. (102)	Sensitivity = 84%, specificity = 94. 4%	Sensitivity = 92%, specificity = 100%
Fang et al. (37)	Sensitivity = 92%, specificity = 91. 7%	Sensitivity = 52%, specificity = 91. 7%
Huang et al. (34)	Sensitivity = 95. 9%, specificity = 95. 2%	Sensitivity = 79. 6%, specificity = 95. 2%,
Cai et al. (103)	Sensitivity = 95. 45%, specificity = 90. 91%	Sensitivity = 72. 72%, specificity = 77. 27%
Wang et al. (38)	Sensitivity = 95. 6%, specificity = 94. 4%	Sensitivity = 77. 8%, specificity = 94. 4%

## 4 Comparative evaluation of mNGS versus other molecular diagnostic techniques

Polymerase chain reaction (PCR) has been introduced into the field of periprosthetic joint infection (PJI) diagnosis due to its rapidity and high sensitivity (40). However, the issue of false-positive results remains a major clinical concern (41, 42). These false positives often arise from various factors such as cross-contamination, non-specific amplification, suboptimal primer design, or improper sample handling. In recent years, multiple studies have focused on reducing the false-positive rate through improvements in detection methodologies and optimization of experimental procedures (43–46). Furthermore, continuous monitoring of melting temperature (47) and integration of culture-based verification (48) are also crucial. These measures are expected to enhance the reliability of PCR-based detection, thereby supporting precision medicine and public health decision-making. Compared with other diagnostic techniques, mNGS also possesses numerous advantages. A primary strength of mNGS is its unbiased sampling capability, enabling broad identification of both known and unexpected pathogens, including novel organisms (49). mNGS can also be coupled with targeted approaches, such as using primers from conserved 16S ribosomal RNA (rRNA) and internal transcribed spacer regions for universal bacterial and fungal detection (50, 51), allowing species-level identification of these organisms. Another advantage of mNGS is its ability to provide strain-level characterization and antimicrobial resistance prediction (52–54). Moreover, the diagnostic accuracy of mNGS significantly surpasses that of conventional PCR. A meta-analysis of 79 studies demonstrated that mNGS exhibits the highest overall diagnostic performance for PJI (39). These findings indicate that while maintaining high specificity, mNGS substantially improves detection rates for PJI, particularly in culture-negative or low-biomass infections. Traditional molecular methods like 16S rDNA PCR show only 73%–86. 5% concordance with mNGS, underscoring the need for clinical correlation to avoid overreliance on any single technique (38, 39). Compared to alternative diagnostic methods, mNGS-guided targeted therapy facilitates faster inflammation control, shorter treatment duration, and potential reduction in hospitalization (55). Furthermore, its diagnostic superiority in complex infections strongly supports broader clinical adoption (Table 2).

**Table 2:** Comparison among mNGS, PCR and standard culture.

Comparison dimension	mNGS	PCR	Standard culture
Core process	Sample treatment → nucleic acid extraction → library construction → high-throughput sequencing → bioinformatics analysis.	Sample treatment → nucleic acid extraction → polymerase chain reaction-sequence specific primers → Electrophoresis/fluorescence detection.	Sample inoculation → selective medium culture → observe the colonies → biochemical identification.
Total time	24–48 h	6–12 h	2–7 d
Detection rang	Unbiased detection: bacteria, fungi, viruses, parasites, etc.	Targeted detection: Only covers specific pathogens within the range of primer design.	Live bacteria dependence: Only growable pathogens can be cultivated (prone to missed detection).
Sample type	Synovial fluid, tissue, ultrasonic lysis buffer.	Applicable to the majority of samples.	Adequate live bacteria (decreased sensitivity after antibiotic use).
Prediction of drug resistance genes	It can directly detect drug resistance genes (genotype–phenotype consistency needs to be verified).	Additional design of drug resistance gene testing (such as mPCR).	Dependent drug sensitivity test (to be completed after culture).

## 5 Pathogen spectrum expansion value of mNGS in PJI

### 5.1 Superiority in polymicrobial infection detection

mNGS demonstrates superior performance over conventional culture methods in identifying polymicrobial infections. Its high-throughput, culture-independent nature provides a novel approach for rapid and accurate diagnosis of PJI, particularly in mixed infections (56, 57). Notably, in polymicrobial infection cases, mNGS achieves a sensitivity of 72. 23%, compared to merely 27. 27% for culture, underscoring its enhanced capability to detect co-infecting pathogens (35, 56, 58). This high sensitivity renders mNGS a powerful tool for diagnosing culture-negative PJI, especially those caused by fastidious pathogens in mixed infections.

### 5.2 Identification of rare and fastidious microorganisms

In PJI, beyond common pathogens like *Staphylococcus aureus* and *Staphylococcus epidermidis*, mNGS has successfully detected clinically significant Gram-positive bacteria that were rarely reported in PJI due to their fastidious growth requirements (59, 60). The application of mNGS enables identification of these challenging-to-culture anaerobic organisms, including Mycoplasma, *Candida parapsilosis*, Brucella, and non-tuberculous mycobacteria (24, 61–64), among other rare pathogens. These bacteria demand specialized culture conditions and exhibit slow growth (65). In addition to these major pathogen groups, mNGS has detected even rarer organisms in PJI (26), such as *Coxiella burnetii* in culture-negative PJI samples where serological testing may initially yield negative results (32, 66). These discoveries significantly expand our understanding of the PJI pathogen spectrum, particularly in detecting rare and fastidious pathogens that conventional cultures often miss (59, 67). These microorganisms span diverse categories, including certain bacteria, fungi, viruses, and parasites (33, 68–71). The implementation of mNGS technology holds critical clinical value for elucidating PJI’s complex etiology, guiding targeted therapy, and improving patient outcomes.

## 6 Clinical applications of mNGS: enhancing diagnosis and guiding targeted therapy

### 6.1 Diagnostic advantages of mNGS

mNGS demonstrates significant time efficiency and superior anti-interference capability compared to culture methods, which typically require several days—or even over a week for certain rare pathogens—to yield results (72). In contrast, mNGS reduces the average detection time to 24–48 h (73, 74). Studies indicate (37, 75) that mNGS can complete testing and result interpretation within 48 h of specimen receipt. This temporal advantage is particularly critical for PJI patients requiring urgent treatment, as it mitigates the adverse effects and antimicrobial resistance risks associated with empirical broad-spectrum antibiotic use (31). Notably, mNGS exhibits enhanced diagnostic performance in patients with preoperative antibiotic exposure (24), attributable to its culture-independent nature, which enables detection of residual pathogen DNA post-antibiotic treatment (31, 37). Furthermore, mNGS maintains 92% sensitivity even in low-volume samples (e. g., 1 mL synovial fluid), whereas traditional culture sensitivity drops to 52% under these conditions (37). Approximately 25% of PJI cases show culture-negative results due to antibiotic pretreatment or rare pathogens, leading to significantly prolonged hospitalization compared to culture-positive cases (61). A multicenter retrospective study comparing targeted therapy based on mNGS results (TM group) versus empirical treatment (EM group) demonstrated that mNGS achieved significantly higher diagnostic positivity rates. The TM group exhibited faster postoperative declines in C-reactive protein (CRP) and erythrocyte sedimentation rate (ESR), with significantly shorter normalization times than the EM group (55). Although mNGS incurs costs, its precision diagnostics reduce the burden of erroneous treatments and improve clinical outcomes—such as avoiding unnecessary revision surgeries or prolonged antibiotic courses—justifying its value.

### 6.2 mNGS-guided treatment strategy optimization

By directly detecting microbial nucleic acid sequences in clinical samples without relying on pathogen cultivation, mNGS effectively identifies pathogens undetectable by conventional methods, thereby directly influencing clinical decision-making. In a case report (76), mNGS analysis of joint aspirate successfully identified *Aggregatibacter aphrophilus* nucleic acid sequences, with subsequent culture confirming its antibiotic susceptibility profile. For acute PJI, pathogen identification via mNGS achieved a 91. 7% success rate in debridement-antibiotics-implant retention (DAIR) procedures (77). Furthermore, a study of patients with unexplained ESR/CRP elevation revealed that while bacterial cultures were negative, mNGS detected bacterial infections in 63. 63% of cases, guiding postoperative antibiotic management and achieving 100% infection-free survival (78). mNGS enables early detection of resistance genes to optimize antibiotic selection and guide combination therapy (26, 79), a capability absent in traditional methods. For instance, polymicrobial infections may require broad-spectrum regimens covering aerobic, anaerobic, and atypical pathogens rather than single antibiotics (80, 81). Research demonstrates (57) that early mNGS-based diagnosis allowed Mycoplasma PJI cases to be cured with antibiotics alone, avoiding surgery. mNGS-guided therapy improved the 2-year cure rate of sinus tract-associated PJI from 55. 6% to 94. 4%, while reducing antibiotic duration by 11. 5 days (58). Although mNGS unit costs exceed culture, its 22% reduction in treatment modifications may lower overall healthcare expenditures (79, 82). These findings confirm mNGS enhances clinical precision by reducing empirical antibiotic misuse and unnecessary surgeries.

### 6.3 Limitations of mNGS and corresponding mitigation strategies

While mNGS technology has significantly enhanced pathogen detection capabilities in PJI—particularly demonstrating irreplaceable value for polymicrobial infections, rare pathogens, and culture-negative cases—its clinical interpretation remains controversial. Key challenges include host DNA interference, ambiguous criteria for false-positive/negative determinations, high costs, and clinical decision-making dilemmas when results conflict with culture findings. Multicenter studies and large-scale clinical validation are urgently needed to clarify its optimal applications and refine implementation pathways.

### 6.4 Technical bottlenecks: sensitivity and specificity limitations

Host DNA depletion, a critical step in enhancing metagenomic sequencing efficiency, requires careful selection based on sample type, characteristics of target microorganisms, and specific application scenarios. Physicochemical methods significantly improve the signal-to-noise ratio (53, 83), while molecular biology-based amplification (84) and bioinformatic filtering (85, 86) offer complementary solutions for low-biomass samples. Furthermore, threshold setting remains highly contentious: some studies propose >10 unique reads as a positive criterion, yet this may lead to under-detection of low-biomass pathogens and over-interpretation of high-abundance commensals (34, 87, 88). Future development should focus on creating more universal host cell lysis reagents, optimizing multiplex molecular enrichment strategies, and establishing standardized performance evaluation frameworks. With continuous technological refinement, the application prospects of mNGS in precision diagnosis and environmental monitoring will broaden considerably.

### 6.5 Interpretation controversies of mNGS results

Pathogens detected by mNGS require clinical correlation to determine their pathogenicity. Current studies primarily evaluate mNGS using fresh PJI samples, typically including synovial fluid, sonicate fluid from prostheses, or tissue (50). Performance varies significantly across sample types, with issues like false positives/negatives observed (28), though systematic comparisons remain lacking. Additionally, detection of multiple pathogens may obscure true causative organisms, complicating interpretation (89, 90). For instance, while mNGS improves polymicrobial detection in immunocompromised patients (91), distinguishing colonization from true infection becomes more challenging. One study reported 47. 1% of mNGS-positive cases were clinically confirmed as colonization (92). For such discrepancies, multiplex PCR may serve as a rapid screening tool (79, 93). A fundamental limitation of mNGS is its inability to differentiate live/dead bacteria. This could be addressed by integrating microbial viability markers (e. g., Precision Run-On Sequencing, PRO-seq) to assess translational activity (94, 95), potentially complementing mNGS in future diagnostic frameworks. While mNGS serves as a valuable adjunct to conventional methods, its inherent limitations necessitate comprehensive clinical contextualization for accurate result interpretation.

### 6.6 Economic and temporal constraints in mNGS implementation

Although mNGS has shortened the turnaround time for some diagnostics (84), its high cost limits routine clinical application, particularly in resource-limited healthcare settings (29). The substantial expenses associated with mNGS primarily arise from sample preparation (96), the sequencing process (79), and the complexity of bioinformatic analysis (25). These technical steps require specialized equipment and trained personnel, contributing to both direct financial burden and time investment (54, 97). Studies indicate (79, 82) that the cost of a single mNGS test is approximately $260, compared to less than $50 for culture. However, evidence regarding the balance between direct detection expenses and indirect savings—such as reduced reoperation rates—remains insufficient, underscoring the need for further research to quantify the overall cost-effectiveness of mNGS.

### 6.7 Absence of unified standards for mNGS implementation

Current mNGS lacks standardized experimental protocols and bioinformatics analysis criteria. Significant variations exist across centers in sample collection volumes for synovial fluid, tissue, or prosthesis sonicate fluid (27, 31, 36), potentially due to differences in DNA extraction methods or sequencing platforms (97). Since mNGS can detect microbial contaminants from samples, processing reagents, or laboratory environments, result interpretation becomes complicated. Even biopsies from theoretically sterile sites may be inadvertently contaminated during routine clinical sampling. Therefore, strict adherence to quality control procedures for reagents and workflows is essential to maintain an aseptic, nucleic acid-free testing environment and prevent cross-contamination that could lead to false-positive results (28, 98, 99). Furthermore, bioinformatics analysis remains unstandardized. For instance, resistance gene detection matched phenotypic resistance in only 37. 5% of culture-positive PJIs (79, 100), with insufficient accuracy limiting its therapeutic guidance value (32, 101). While international consensus meetings recommend incorporating mNGS as an adjunct criterion in MSIS diagnostic standards, most supporting evidence remains level II-III (28, 39). Future efforts should establish expert consortiums to develop standardized mNGS operational guidelines for PJI.

## 7 Discussion

mNGS is reshaping the diagnostic paradigm for PJI, with its core value lying in overcoming the limitations of traditional culture methods to achieve rapid and comprehensive pathogen identification. However, its clinical application remains constrained by issues such as technical performance (inability to determine whether detected sequences originate from live or dead pathogens), cost-effectiveness, and lack of standardization (absence of standardized procedures and criteria to prevent nucleic acid contamination from specimen collection through processing and environmental exposure). At this stage, the technology cannot yet fully replace conventional microbial culture. Targeted sequencing (tNGS) and nanopore sequencing (mONS) represent important directions for the development of mNGS. tNGS offers lower costs, shorter turnaround times, and the ability to simultaneously detect resistance genes. Nanopore sequencing enables real-time pathogen identification and resistance analysis, although the issue of host DNA interference remains to be resolved. Furthermore, the combination of multiplex PCR (mPCR) with mNGS can shorten pathogen detection time, making it suitable for rapid screening. Currently, mNGS should serve as a complementary approach to traditional culture, with a focus on cases of culture-negative PJI (CN-PJI), patients with preoperative antibiotic exposure, and suspected polymicrobial infections. The establishment of standardized protocols is a core prerequisite for its broader clinical adoption. Future research should focus on three major aspects: first, developing multicenter standardized testing protocols based on sonicate fluid from explanted prostheses to address operational heterogeneity; second, creating efficient host DNA depletion techniques to improve pathogen nucleic acid capture efficiency; and third, advancing clinical validation of mNGS-guided personalized antibiotic therapy and exploring its translational potential in resistance prediction. Optimizing sample processing, establishing tiered reporting standards, and integrating multimodal diagnostic results are essential to balance detection breadth with clinical utility. Dedicated to establishing a more unified definition and diagnostic criteria for PJI, through the accumulation of high-quality evidence from multicenter studies and large-sample validation can mNGS be integrated into the standard PJI diagnosis and treatment framework. This integration will ultimately achieve the goals of improving patient outcomes, curbing antibiotic misuse, and establishing a closed-loop mNGS-driven system for rapid diagnosis and targeted therapy in PJI. mNGS holds promise to advance from an auxiliary diagnostic tool to a new gold standard for pathogen diagnosis in PJI.
